# Antimicrobial activity of natural products against *Helicobacter pylori*: a review

**DOI:** 10.1186/s12941-014-0054-0

**Published:** 2014-11-19

**Authors:** Bruna Vidal Bonifácio, Matheus Aparecido dos Santos Ramos, Patricia Bento da Silva, Taís Maria Bauab

**Affiliations:** Department of Biological Sciences, School of Pharmaceutical Sciences, São Paulo State University, Rodovia Araraquara-Jaú, km 01, Araraquara, SP CEP 14801-902 Brazil; Department of Drugs and Medicine, School of Pharmaceutical Sciences, São Paulo State University, Rodovia Araraquara-Jaú, km 01, Araraquara, SP CEP 14801-902 Brazil

**Keywords:** *Helicobacter pylori*, Natural products, Antimicrobial activity, *Helicobacter pylori*, Produtos naturais, Atividade antimicrobiana

## Abstract

Throughout the genetic and physiological evolution of microorganisms, the microbiological sciences have been expanding the introduction of new therapeutic trials against microbial diseases. Special attention has been paid to the bacterium *Helicobacter pylori*, which induces gastric infections capable of causing damage, ranging from acute and chronic gastritis to the development of gastric cancer and death. The use of compounds with natural origins has gained popularity in scientific research focused on drug innovation against *H. pylori* because of their broad flexibility and low toxicity. The aim of this study was to describe the use of natural products against *H. pylori* in order to clarify important parameters for related fields. The study demonstrated the vast therapeutic possibilities for compounds originating from natural sources and revealed the need for innovations from future investigations to expand the therapeutic arsenal in the fight against *H. pylori* infection.

## Additional non-English language - Portuguese

No decorrer da evolução genética e fisiológica dos micro-organismos, as ciências microbiológicas encontram-se em expansão, preocupando-se em introduzir novas triagens terapêuticas frente às afecções microbianas, com especial atenção àquelas adquiridas pela bactéria *Helicobacter pylori,* sendo esta frequentemente envolvida nas infecções gástricas capazes de causar dano ao organismo humano que vão desde gastrites agudas e crônicas ao desenvolvimento de neoplasias gástricas, levando o paciente ao óbito na maioria das vezes. Neste sentido, o uso de compostos de procedência natural ganha destaque em pesquisas científicas voltadas a inovação medicamentosa contra o *H. pylori*, visto a vasta flexibilidade de investigação terapêutica e a característica de baixa toxicidade. Este trabalho teve como objetivo descrever sobre o uso de produtos naturais frente ao combate do *H. pylori*, a fim de esclarecer parâmetros importantes para todas as áreas afins. O estudo demonstrou quão vasta são as possibilidades terapêuticas exercidas por compostos oriundos de fontes da natureza e, verificou a necessidade de inovações voltadas ao aprofundamento de investigações com o intuito de ampliação do arsenal terapêutico frente ao combate do micro-organismo em questão.

## Introduction

The relationship between *Helicobacter pylori* and acquired resistance to various drugs from conventional therapy is of worldwide concern. Several global consensus sessions have been conducted to ensure that medical guidelines are consistently updated on various issues involving the management of infection [1]. The first “Asian-Pacific *H. pylori* Consensus Conference” took place in Singapore in August 1997, and since then, new scientific information concerning the treatment of infection by this bacterium has been published along with updates from conferences in North America and Europe [2]. With antibiotic resistance reaching a crisis point in countless medical and scientific centers around the world and the growing resistance rate affecting communities, there is an urgent need to restore the arsenal of antimicrobial agents [3]. In addition to the high rate of resistance observed in conventional therapy, the chances of abandoning the treatment are large, mainly due to side effects or recurrence of infection. Therefore, it is extremely important to search for new therapeutic sources with anti-*Helicobacter pylori* action. Currently, plants are viewed as the main source for the discovery of new compounds [4].

The use of natural products in the therapeutic management against diseases caused by microorganisms such as *H. pylori* presents advantages over drugs derived from synthetic sources. This is due to the low side effects of these drugs when their toxicological and pharmacological activity is compared to those obtained from industrial sources. In addition to the lower toxicity, areas such as gastroenterology and bacteriology have shown remarkable interest in the pharmacological activities that natural products have against infectious agents. The health sciences have been increasingly concerned with the exacerbated growth in the number of *H. pylori* strains multi-resistant to antibiotics currently used in clinical practice. This may be explained by the indiscriminate use of drug therapies leading to a higher incidence of failures in treatment [5-7].

Given the numerous benefits provided by natural products according to the literature, this paper presents a bibliographical survey of reports of antimicrobial action exerted by them against *Helicobacter pylori* published between the years 1996 and 2013, with the purpose of detailing publications of great importance to the scientific community.

## Review

### General aspects

Identified in Australia (1982) by the researchers Barry Marshall and J. Robin Warren, *Helicobacter pylori* was isolated from gastric biopsy specimens from patients with chronic gastritis and peptic ulcers [1,4,8]. It was difficult to demonstrate that this pathogen was really the major cause of gastritis, peptic ulcer and gastric cancer; therefore, to convince his teammates and the entire audience, Barry Marshall took a suspension of the pathogen and confirmed by means of Koch’s postulates that *H. pylori* was the major cause of gastric pathologies [9].

*H. pylori* is classified as a Gram-negative, spiral and microaerophilic bacterium that specifically colonizes the gastric mucosa and affects more than half of the world’s population. In most cases, it is acquired in childhood and, if left untreated, often persists into adulthood. Its helical shape favors movement driven by flagella, causing a disruption in the protective stomach lining. In association with the release of cytokines and the chronic inflammatory process, this disruption can develop into more serious and acute diseases, such as chronic gastritis, peptic ulcer and gastric cancer [10-12].

*H. pylori* is considered one of the most common causes of infection globally. The World Health Organization (WHO) ranked the pathogen as a Class I carcinogen for gastric cancer based on the results of epidemiological studies demonstrating its ability to induce carcinogenesis without the administration of co-carcinogens [2,10,13]. Based on this, *H. pylori* quickly became the subject of several studies in various health areas ranging from microbiological to histological, epidemiological, immunological, and ecological [1,8].

**Figure 1:**
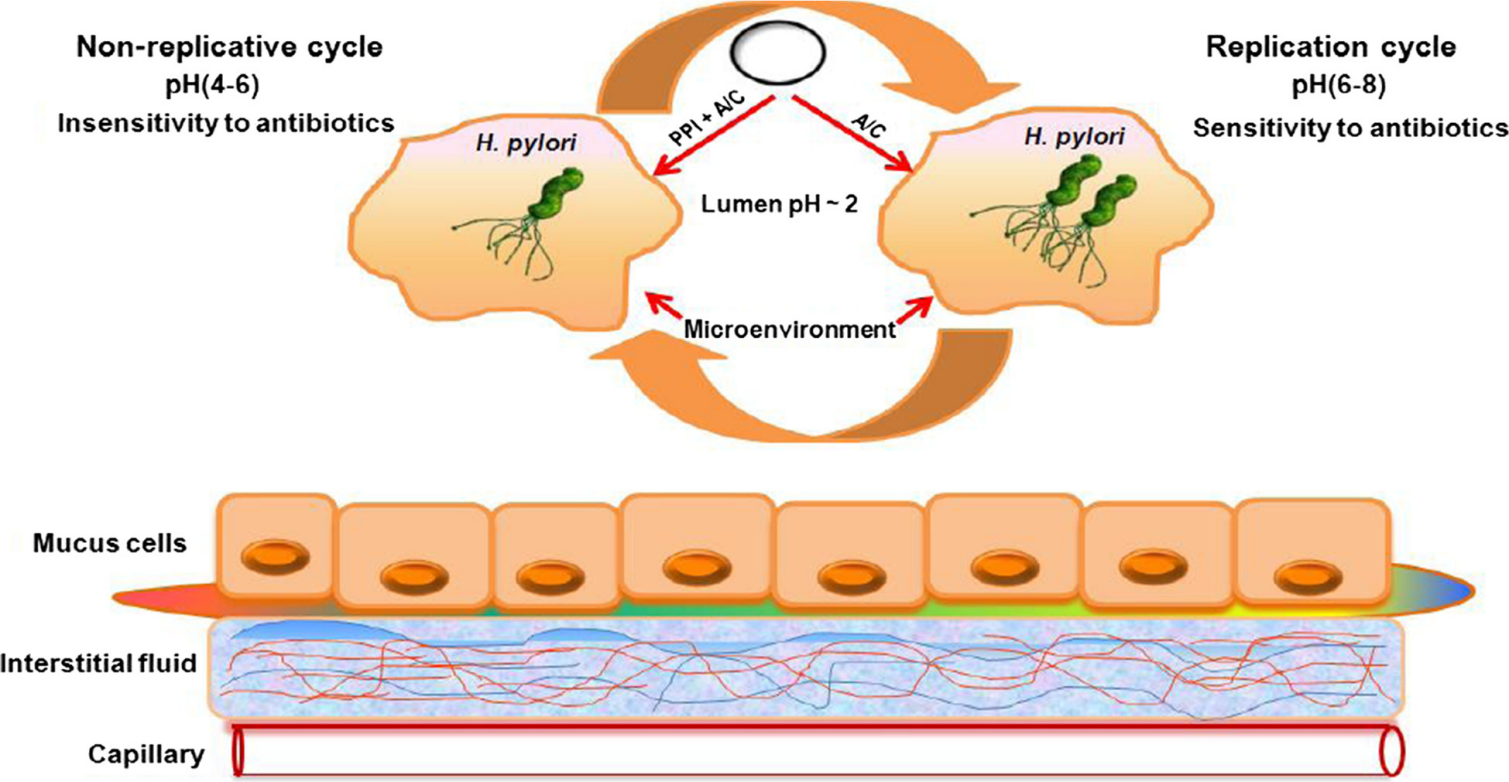
**United of replication of** ***H. pylori*** **(antibiotic sensitivity), and a non-replicative state (antibiotics insensitivity).** Cycles occurring according to the pH in the microenvironment. PPI: proton pump inhibitor, A: amoxicillin, C: clarithromycin [14].

First-line treatment consists of triple and quadruple therapies. Triple therapy includes a proton pump inhibitor (PPI) such as omeprazole combined with two antibiotics, usually amoxicillin and clarithromycin. Quadruple therapy contains one additional drug containing bismuth. Sequential therapy is a simple dual therapy regimen including a PPI plus amoxicillin given for the first 5 days, followed by triple therapy including a PPI, clarithromycin and tinidazole (all twice daily) for the remaining 5 days [14]. Triple therapy has an efficacy of 75% but is expensive and associated with drug resistance and numerous side effects, such as observed with allergy and cardiovascular drugs, while sequential therapy has an efficacy of 90% or greater. However, this approach may fail because bacteria can oscillate between a replicative state (microorganism remains susceptible to the antibiotic) and a non-replicative state (microorganism becomes resistant phenotype) according to the pH of its microenvironment. The bacteria cannot enter the replicative cycle when the pH is between 4.0 and 6.0, and the microorganisms are difficult to eradicate when they assume the phenotypically resistant state (Figure 1) [15,16].

### Virulence factors

Currently, urease, vacuolizing cytotoxin and pathogenicity gene products (cagPAI - cag pathogenicity island) are considered to be the major virulence factors of the pathogen [17]. Some strains of *H. pylori* have a pathogenicity island (PAI) known as the cytotoxin related to gene A (cag A), which in combination with the vacuolizing cytotoxin (Vac A) results in a higher degree of virulence [12].

The pathogenicity of the bacteria is increased by the presence of PAI; thus, *H. pylori* can be classified as cag + or cag-. The proteins encoded by the cag PAI are responsible for inducing secretion of interleukin-8 (IL-8), remodeling the surface of epithelial cells and forming the pedestal. *H. pylori* can be divided into two types: type I expresses cagA^+^ associated with cytotoxin VacA, whereas type II does not express *cagA* associated with *vacA. VacA* has the ability to induce large cytoplasmic vacuoles in eukaryotic cells, which can lead to cell death [12].

*H. pylori* interacts closely with epithelial cells, causing a variety of responses via diverse molecular interactions such as the release of cytokines capable of activating inflammatory cells. The type IV secretion system (T4SS) transfers the CagA oncoprotein into the host cell cytoplasm, where it becomes phosphorylated and affects different cellular processes. This is considered the direct contact of T4SS with the epithelial cell membrane; however, it is believed to be primarily responsible for the induction of IL-8 and other host immune responses. The α5β1 epithelial integrin has been implicated as one of the host receptors involved in this activity. However, the expression of these integrins is limited to the basolateral membrane. A monolayer *in vitro* model of epithelial cells with narrow junction integrity and measurable barrier function would facilitate the study of *H. pylori*-host cellular interactions and allow detailed examinations of the molecular events that occur apical and basolaterally during infection by the pathogen [10].

BabA is a protein present in the membrane of the bacteria that ensures adhesion to the epithelial cell. These circular fibrillar structures cover the microorganism and hamper its elimination by peristaltic movements, allowing the secretion of factors that attract and stimulate inflammatory cells [12].

It is believed that during the course of infection, there is an increase in gastric acid and gastrin secretion. This relationship has drawn the attention of many researchers from different scientific fields seeking therapeutic alternatives that may behave favorably given all the factors involved [18].

Because the gastric mucosa is considered to be the main habitat of *H. pylori*, its survival in an acidic medium is of the utmost importance. This survival is ensured by the urease enzyme, which is capable of hydrolyzing the urea present under physiological conditions in acidic medium. The production of ammonia that occurs through this hydrolysis acts as a receptor for H^+^ ions and consequently generates a neutral pH in the intracellular environment. Urease and ammonia promote destabilization of the mucus layer, leading to formation of lesions on the lining cells. Furthermore, urease may also be involved in the activation of neutrophils, monocytes and the immune system, resulting in local inflammatory lesions [12,19].

The catalytic power of proteases is essential for gastric colonization and *H. pylori* survival. The serine protease HtrA has been found in most infected individuals and is thus considered to be essential for the survival of the bacteria. Lysosomal cathepsins and metalloproteases are abundant proteases found during the early stages of *H. pylori*-mediated pathogenesis. Therefore, this class of protease enzymes may play a functional role in the carcinogenesis of the stomach [20].

The occurrence of monochloramine is also related to gastric injuries detected in the presence of *H. pylori*. This derivative creates large amounts of ammonia [19] and activates neutrophils that produce hypochlorous acid to react with the amino acid taurine, forming taurine-chloramine. Chloramines can be either short or long term and have hydrophilic or lipophilic characters. The lipophilic character is long term and exhibits a better degree of diffusion across the plasma membrane, causing oxidative damage to various biomolecules [21,22].

### Major pathologies associated with the presence of *Helicobacter pylori*

The *Helicobacter pylori* bacterium is characterized as the causative agent of various types of gastric pathologies. It is often involved in cases of chronic gastritis, functional dyspepsia, peptic or duodenal ulcers, and cancer or gastric lymphomas. Because it can survive in acidic environments, it remains intact in the stomach and promotes the destruction of the gastric mucosa. This makes the organ sensitive and vulnerable to triggering of ulcerative lesions and blocks the sterilization of food, producing failures in the digestion process. Patients who have chronic gastritis have a higher risk for the development of peptic ulcers and carcinomas with increased severity. This risk is especially problematic in individuals with chronic multifocal atrophic gastritis, a type of autoimmune disease in which antibodies attack the mucosal lining of the stomach, causing thinning and loss of many or all of the cells that produce acid and enzymes. This disorder is most commonly observed in the elderly, although there is also a tendency for it to occur in people who have had part of their stomach extirpated (a surgical procedure called partial gastrectomy). Atrophic gastritis may cause pernicious anemia because it interferes with the absorption of vitamin B12 from food. The symptoms of gastritis in general are burning, abdominal pain, loss of appetite, nausea, vomiting, feeling of satiety and gastrointestinal bleeding. Deficiency in absorption of elements and vitamins may also occur, causing weakness and diarrhea [23-25].

Currently, dyspeptic syndrome or functional dyspepsia is characterized as a common universal problem presenting several disorders, especially peptic diseases determined by chloridropeptic dysfunction; these include gastroesophageal reflux disease and gastroduodenal peptic ulcer. Although occurring with less intensity, infection with *H. pylori* is directly linked to the development of this pathology, with a significant reduction or complete elimination observed when the patient is subjected to treatment with drugs to combat the bacterium [26-28].

Among all the diseases caused by this pathogen, the greatest focus is given to the development of stomach cancer. This type of pathology is ranked fourth in the world and is directly related to both the progress of the malignant action of the microorganism and genetic predispositions.

Estimates of incidence and mortality due to cancer conducted by the National Cancer Institute (INCA) show that 20,090 new cases of gastric cancer were forecast for 2012 in Brazil, with 63% in men. Approximately 65% of patients diagnosed with gastric cancer are more than 50 years old, with a peak incidence at approximately 70 years of age [28].

According to Antunes et al. [29], despite the efforts made by various areas in health (such as gastroenterology and microbiology) for the decline and prevention in prevalence of microorganisms in the host, infection with *H. pylori* remains the greatest risk factor for the development of gastric cancer, increasing the incidence risk for this cancer approximately six-fold. With worldwide prevalence estimated at between 50 and 90%, this type of cancer frequently occurs in developing countries. In countries such as China with high incidence rates of *H. pylori,* there is an intense parallelism with gastric cancer. Most malignant tumors occurring in the stomach environment are of adenomatous origin, classified according to Lauren’s classification into two main histological types: well-differentiated or intestinal, and diffuse or poorly differentiated [30]. According to Argent et al. [31], incidences of gastric cancer are associated with the presence of *H. pylori* with a positive profile for the CagA + gene, but factors such as diet and genetic polymorphisms are directly related to the onset of the pathology and degree of intensity. Moreover, the combination of polymorphisms of proinflammatory cytokines and infection arising from strains with high virulence profiles influences the malignant carcinogenesis process. Silva et al. [32] confirmed the association of halitosis with the presence of *Helicobacter pylori*, especially when an imbalance occurs in the oral ecosystem due to a decrease in salivary flow or the presence of periodontal disease. Although the stomach may be considered the main reservoir of bacteria in men, oral cavities may be considered as the second, thus allowing the isolation of *H. pylori* in dental biofilms, saliva and gingival sulcus.

### Natural extracts and essential oils used against *Helicobacter pylori*

The various activities presented by natural products are directly related to the presence of bioactive compounds. In this review we will focus on flavonoids, which are responsible for many of them. Several studies have sought the mechanism of action by which flavonoids contribute to anti-*H. pylori* activity. Several studies have shown that compounds from the flavonoid and chalcone classes inhibit the enzyme urease, which is secreted by the bacterium during infection to ensure its survival in the acid pH of the stomach. This may to some extent explain the *in vivo* activity of the quercetin flavonoid against *H. pylori* in Guinea pigs, as well as the efficacy of sofalcone (a derivative of chalcone) in various drugs that comprise the treatment of infection by this pathogen. Nevertheless, other mechanisms can also explain the activity of flavonoids, such as VacA neutralization and interference with toll-like receptor 4 signaling (TLR4). It is also possible that certain flavonoids may exert direct activity against *H. pylori* or act synergistically with antibiotics used in conventional therapy [3].

Several natural products have demonstrated antimicrobial activity against *H. pylori*, and for centuries, a wide variety of plants and substances derived from alternative sources have been used in the treatment of gastrointestinal disorders [4]. Data presented in world literature demonstrates significant results obtained from plant extracts (Table 1) against *H. pylori*, generating significant contributions and increasing the therapeutic arsenal used in infectious cases.

**Table 1:** **Anti-** ***Helicobacter pylori*** **activity of plants**

**Scientific name**	**Family**	**Plant part used**	**Material**	**Type of** ***Analysis***	**Ref.**
*Feijoa sellowiana* (Berg.) Burret.	Myrtaceae	Fruit	Acetone Extract	*In vitro*	[34]
*Strychnos pseudoquina* A. St. Hil.	Loganiaceae	Leaves	Methanol extract/alkaloid enriched fraction	*In vitro* and *in vivo*	[35]
*Bixa orellana* L.	Bixaceae	Seed	Ethanol Extract	*In vitro*	[4]
*Chamomilla recutita* L.	Asteraceae	Inflorescence	Ethanol Extract	*In vitro*	[4]
*Ilex paraguariensis* A. St.-Hil.	Aquifoliaceae	Green leaves	Ethanol Extract	*In vitro*	[4]
*Ilex paraguariensis* A. St.-Hil.	Aquifoliaceae	Roasted Leaves	Ethanol Extract	*In vitro*	[4]
*Malva sylvestris* L.	Malvaceae	Inflorescence and leaves	Ethanol Extract	*In vitro*	[4]
*Plantago major* L.	Plantaginaceae	Above-ground parts	Ethanol Extract	*In vitro*	[4]
*Rheum rhaponticum* L.	Polygonaceae	Root	Ethanol Extract	*In vitro*	[4]
*Punica granatum* L*.*	Punicaceae	Peel	Methanol extract	*In vitro*	[36]
*Juglans regia* L.	Juglandaceae	Fruit ridge	Methanol extract	*In vitro*	[36]
*Davilla elliptica* St. Hil.	Dilleniaceae	Leaves	Methanol extract	*In vitro*	[37]
*Davilla nítida* (Vahl.) Kubitzki.	Dilleniaceae	Leaves	Methanol extract	*In vitro*	[37]
*Byrsonima fagifolia* Niedenzu (IK.)	Malpighiaceae	Leaves	Methanol extract	*In vitro*	[38]
*Qualea parviflora* Mart.	Vochysiaeceae	Bark	Methanol extract	*In vitro* and *in vivo*	[39]
*Hancornia speciosa* Gomez	Apocynaceae	Bark	Hydroalcoholic extract	*In vitro* and *in vivo*	[40]
*Byrsonima intermedia* A. Juss.	Malpighiaceae	Leaves	Methanol extract	*In vitro* and *in vivo*	[41]
*Larrea divaricata* Cav*.*	Zygophyllaceae	Leaves and tender branches	Aqueous extract	*In vitro*	[42]
*Hericium erinaceus*	Hericiaceae	Mushrooms	Ethanol Extract	*In vitro*	[43]
*Allium sativum* L.	Liliaceae	Bulb	Aqueous extract	*In vitro*	[44]
*Pistacia lentiscus* (L.) var. chia (Duham)	Anacardiaceae	Mastic gum	Extract/acid and neutral fractions	*In vitro* and *in vivo*	[51]

According to Brazilian Pharmacopoeia [33], an extract can be defined as a preparation of liquid, solid or intermediate consistency obtained from animal or vegetable material. The component used to prepare extracts may undergo pretreatment, such as inactivation of enzymes, milling or degreasing. The extract can be prepared by percolation, steeping, or other suitable and validated methods, using ethanol, water or another solvent, which varies according to the needs of each material used. After this process, undesirable materials can be eliminated.

Basile et al. [34] demonstrated *in vitro* that the acetone extract of the fruits of *Feijoa sellowiana* (Berg.) Burret. (Myrtaceae) has significant anti-*H. pylori* activity. This activity can be explained by the presence of the flavone compound, which when measured alone had higher activity against this bacterium compared to the metronidazole (0.5 μg/mL) control.

Bonamin et al. [35] evaluated the healing process and anti-*H. pylori* activity mediated by a methanol extract (ME) and an alkaloid-enriched fraction (EAE) of *Strychnos pseudoquina* A. St. Hil. (Loganiaceae). Administration over 14 days in rats with chronic gastric ulcers induced by 5% acetic acid (an experimental model that accurately reflects human gastrointestinal disease) showed that EAE significantly reduced the edge of the internal (42%) and external (38%) injury areas using microscopic analysis. Animals treated with EAE exhibited stimulation of a few proliferation factors through increase of the height of the epithelial regeneration area and expression of PCNA in the nucleus. The number of vessels in the gastric mucosa of rats treated with EAE was significant increased (four times more than the treatment vehicle) over vessels that stimulate cell proliferation in the scarred region. These results suggest that the vascularization coating in the ulcerative region is involved with the healing action of the alkaloid fraction of *S. pseudoquina*. The minimum inhibitory concentration (MIC) of 75 mg/mL from EAE showed effective *in vitro* anti-*H. pylori* activity. EAE was also very effective in the superoxide dismutase release process, which is an important protective factor against bacterial agents.

Cogo et al. [4] determined whether the use of traditional medicinal plants used in the treatment of gastrointestinal diseases actually presented pharmacological effects or if they are simply based on popular use. Within this context, extracts obtained from *Bixa orellana* L. (Bixaceae), *Chamomilla recutita* L. (Asteraceae), *Ilex paraguariensis* A. St.-Hil. (Aquifoliaceae), *Malva sylvestris* L. (Malvaceae), *Plantago major* L. (Plantaginaceae) and *Rheum rhaponticum* L. (Polygonaceae), all commonly used in the treatment of gastrointestinal diseases, were evaluated for their anti-*H. pylori* activity against standard (ATCC) and clinical strains. The results showed that the extracts obtained from *B. orellana* L., *C. recutita* L. *I. paraguariensis* A. St.-Hil and *M. sylvestris* L. inhibited the *in vitro* growth of *H. pylori*.

Hajimahmoodi et al. [36] evaluated the *in vitro* anti-*H. pylori* activity of methanol extracts from 23 medicinal plants used in the treatment of gastrointestinal disorders. Plants were selected on the basis of traditional medicinal practices by the Iranian community. Among these, only the extracts of *Punica granatum* L. (Punicaceae) and *Juglans regia* L. (Juglandaceae) exhibited high activity against *H. pylori* strains, with inhibition zones of 39 and 16 mm, respectively, according to the agar diffusion technique.

Kushima et al. [37] studied methanol extracts (ME) from the leaves of *Davilla elliptica* St. Hil. (Dilleniaceae) and *Davilla nitida* (Vahl.) Kubitzki. (Dilleniaceae) to examine their anti-ulcerogenic, immunological and anti-*H. pylori* activities. Both extracts protected the gastric mucosa, although *D. nitida* (MIC 125 μg/mL) showed better activity compared to *D. elliptica* (MIC 250 μg/mL). The activity demonstrated is probably explained by the greater quantities of components such as terpenes, flavonoids, tannins, and other compounds present in *D. nitida*.

Lima et al. [38] observed that in addition to healing and antidiarrheal properties, a methanol extract of leaves of *Byrsonima fagifolia* Niedenzu (IK.) (Malpighiaceae) presented antibacterial activity against standard strains of *Escherichia coli, Staphylococcus aureus* and *Helicobacter pylori,* with an MIC for the last two microorganisms of 250 μg/mL.

Mazzolin et al. [39] assessed the gastro-protective, anti-diarrheal, anti-hemorrhagic and mutagenic potential of a methanol extract from the bark of *Qualea parviflora* Mart., a plant belonging to the Vochysiaceae family native to the Brazilian savannah region. This plant is ethnopharmacologically reputed for its important properties in gastro-pathogenic cases and showed remarkable antimicrobial activity when tested against *H. pylori*. Thus, with an MIC of 75 μg/mL, the extract was classified as promising for the inhibition of microorganism. Based on the relevance of the results, methods were developed to assess its gastro-protective potential, with innovative results that expanded the therapeutic and prophylactic possibilities of the herbal compound.

Other studies have also focused on the gastro-protective action of plant products based on ethnopharmacological beliefs of anti-hypertensive properties, treatment of gastric ulcers, inflammatory diseases and stomach disorders. For example, Moraes et al. [40] demonstrated the anti-*H. pylori* potential of the hydroalcoholic extract originating from the bark of *Hancornia speciosa* Gomez (Apocynaceae), a medium-sized tree popularly known as “mangabeira” found in the Brazilian savannah region, using *in vivo* and *in vitro* approaches. The authors observed that the extract was able to inhibit bacterial growth at an MIC of 125 μg/mL using an *in vitro* approach. In the experimental *in vivo* model, the extract was shown to be effective in gastroprotective and antiulcer performance. The same inhibition values were obtained by Santos et al. [41] when investigating the antimicrobial profile of the methanol extract of leaves from *Byrsonima intermedia* A. Juss. (Malpighiaceae) against the same strain. The analysis was performed by means of *in vitro* screening and *in vivo* models from rodent carriers of duodenal ulcer caused by various agents, such as non-steroidal anti-inflammatory drugs and chemical solvents; moreover, a significant antidiarrheal potential was shown when the extract was assessed against intestinal motility.

Stege et al. [42] showed the anti-*H. pylori* action of the aqueous extracts from *Larrea divaricata* Cav. (Zygophyllaceae) against strains highly resistant to conventional drugs. A standard strain (ATCC) and six strains of clinical origin were used, all resistant to clarithromycin and four resistant to metronidazole. The plant extract exhibited activity against all strains.

There has been a significant increase in the consumption of mushrooms by the general population in recent years. Mushrooms have been gaining ground in the culinary world because they have an exotic taste, are highly nutritious and are a source of protein and minerals. The scientific community has been searching for the applicability of these products given their use against pathogenic microorganisms. Shang et al. [43] determined the activity of 14 ethanol extracts of Chinese mushrooms used for food and prophylactic purposes against strains of *Helicobacter pylori* from standard (ATCC) and clinical sources obtained from gastric biopsies of patients with gastric ulcers. All of the extracts were effective against all strains used in the study. Furthermore, the presence of antibodies against *H. pylori* was observed from specific tests for this purpose.

Cellini et al. [44], Sivam et al. [45], and Canizares et al. [46] highlighted the activity of the aqueous extract of garlic against *H. pylori.* A significant synergistic effect was observed by Jonkers et al. [47] with the aqueous extract of garlic and the drug omeprazole, which showed a significant increase in activity compared to results obtained by each component separately. Pharmacokinetic interactions between the extract and the drug may occur during the processes of absorption, distribution, metabolism and excretion. In the case of interactions involved in the absorption process, there may be a decrease or increase in the amount of drug absorbed or in the rate of absorption, resulting in a decrease or increase in the intensity of the pharmacological effect obtained. Phytochemicals present in the extract may also interact with ATP-dependent protein transporters, such as intestinal P-glycoprotein or other proteins that facilitate drug efflux, thereby changing its bioavailability. However, interactions between drugs and plant extracts reported in the literature include alterations in the metabolism of the drug. These effects are mostly due to compounds that cause induction or inhibition of the enzymes responsible for oxidative metabolism belonging to the cytochrome P450 (CYP) family, the main system used to eliminate drugs from the body [48-50].

The resin of *Pistacia lentiscus* (L.) var. chia (Duham), an evergreen shrub belonging to the family Anacardiaceae uniquely cultivated in southern Chios, is known as mastic. Mastic gum has exhibited anti-*H. pylori* activity against several gastrointestinal diseases. Paraschos and co-workers [51] studied the *in vitro* and *in vivo* activities of Chios mastic gum extracts and its constituents against *H. pylori*. The active mastic constituents were obtained by separation of total mastic extract without polymer into an acidic and a neutral fraction. Both fractions were characterized by nuclear magnetic resonance and mass spectroscopy to elucidate the structure of the components. The major triterpenic acids were found in the acid fraction, and triterpenic alcohols and aldehydes composed the neutral fraction. The *in vitro* results using the mastic total extract, fractions and pure compounds showed that the most active extract was the acid fraction, presenting a minimum bactericidal concentration of 0.139 mg/ml, while isomasticadienolic acid was considered the most active pure compound (minimum bactericidal concentration of 0.202 mg/ml). Approximately 1 month after being infected with *H. pylori*, mice were treated over a 3-month period with extract diluted in ethanol and then dissolved in water. These *in vivo* results showed a statistically significant reduction in *H. pylori* viable counts in the group treated with total mastic extract without polymer. PCR, serology, *H. pylori* culture and histopathologic evaluation of the gastric mucosa confirmed these results, indicating that mastic gum may be effective in reducing *H. pylori* colonization.

Other studies of great importance are directed towards the use of essential oils (Table 2) for the treatment of diseases caused by pathogenic and opportunistic microorganisms, such as the use of essential oils in the search for new compounds endowed with therapeutic potential.

**Table 2:** **Anti-** ***Helicobacter pylori*** **activity of essential oils**

**Scientific name**	**Family**	**Type of** ***Analysis***	**Ref.**
*Allium sativum* L.	Liliaceae	*In vitro*	[56]
*Nepeta camphorata* L.	Lamiaceae	*In vitro*	[57]
*Nepeta argolica ssp.* dirphya	Lamiaceae	*In vitro*	[57]
*Cymbopogon citratus* (DC) Stapf.	Poaceae	*In vitro* and *in vivo*	[58]
*Cupressus sempervirens* L.	Cupressaceae	*In vitro*	[58]
*Juniperus communis* L.	Cupressaceae	*In vitro*	[58]
*Melaleuca alternifólia,* Cheel	Myrtaceae	*In vitro*	[58]
*Aloysia citrodora* Palàu	Verbenaceae	*In vitro*	[58]
*Ocimum basilicum* L.	Lamiaceae	*In vitro*	[58]
*Mentha piperita* L.	Lamiaceae	*In vitro*	[58]
*Origanum marjorana* L.	Lamiaceae	*In vitro*	[58]
*Eucalyptus globulus* Labill	Myrtaceae	*In vitro*	[58]
*Ravensara aromatica* Sonnerat	Lauraceae	*In vitro*	[58]
*Citrus limonum* Risso	Rutaceae	*In vitro*	[58]
*Rosmarinus officinalis* L.	Lamiaceae	*In vitro*	[58]
*Lavandula latifolia* Medik*.*	Lamiaceae	*In vitro*	[58]
*Myrtus communis* L.	Myrtaceae	*In vitro*	[59]
*Citrus lemon*	Rutaceae	*In vitro*	[61]

Essential oils or scents are aromatic compounds found in different plant organs. They are also called volatile oils or ethereal oils, as they have a high degree of evaporation when exposed to air at room temperature; it is this feature that confers the significant odor to plants, both for attraction of pollinators and as insect and herbivore repellents [52-54]. These compounds have emerged in the medical field by presenting antimicrobial activities of extreme value with regards to drug action against pathogenic or opportunistic microorganisms, including antifungal and antibacterial action. Because the compounds have a complex constitution, the profile exerted against microorganisms is directly related to this feature; for example, the presence of terpenes, which are secondary metabolites of plants with interesting therapeutic properties, have been studied by the scientific community in recent years. The antimicrobial activity demonstrated by terpenes is attributed to their interference with the integrity and functioning of the cell membrane through induction of changes in membrane potential, loss of cytoplasmic material and inhibition of the respiratory chain [54,55].

Studies with the aim to elucidate the anti-*Helicobacter pylori* profile presented by essential oils have been developed in recent years because of the need for further drug options in the treatment of disorders arising from this type of infection. This fact is justified by the increased number of strains resistant to the standard drug therapy used in clinical practice, such as clarithromycin, the drug most frequently used as a therapeutic agent, and the lack of an ideal drug regimen with 100% safe applicability [8].

To broaden the knowledge of the anti-*H. pylori* potential of essential garlic oil, Otha et al. [56] tested compounds isolated from crude oil and obtained MIC values ranging from 10 to 25 μg/mL.

Other antimicrobial profiles of essential oils are reported in the literature. For example, Kalputzakis et al. [57] observed antibacterial action against clinical *H. pylori* strains extracted from biopsies performed on adults and children from two species of plants from the Nepeta genus, *Nepeta camphorata* L. and *Nepeta argolica ssp. dirphya*, belonging to the Lamiaceae family. The profile established by the essential oils of the two species was shown to be relevant, with minimum inhibitory concentrations (MIC) of 128 and 64 μg/mL for *N. camphorata* and *N. argolica* ssp. *dirphya*, respectively. Four compounds of the two species were also isolated and tested, presenting MICs ranging from 16 to 64 μg/mL for the same strains. The results obtained from the isolated substances confirm that flavonoids are mainly responsible for the antimicrobial activity for products derived from plant sources, as these substances were characterized as flavonoid derivatives.

Ohno et al. [58] reported the action of 13 essential oils against strains of *H. pylori* from clinical and standard origin (ATCC). The study found activity against all strains tested with oils extracted from *Cupressus sempervirens, Juniperus communis Melaleuca alternifolia, Lippia citriodora, Ocimum basilicum, Mentha piperita, Origanum majorana, Eucalyptus globulus., Ravensara aromatica, Citrus limonum, Cymbopogon citratus, Rosmarinus officinalis and Lavandula latifolia*. The same sensitivity profile was observed by Deriu et al. [59] in 2007, who investigated the activity of the essential oil of *Myrtus communis* L. against ten clinical isolates of *Helicobacter pylori* with a resistant profile for triple therapy with metronidazole, clarithromycin and levofloxacin.

The essential oil of *Sicilian lemon* (*Citrus lemon* Burm. Rutaceae) is classified as a potentially promising product against gastrointestinal diseases [60]. A study regarding its antimicrobial potential against *Helicobacter pylori* by Rozza et al. [61] revealed a minimum inhibitory concentration of 125 μg/mL. Furthermore, the authors performed a phytochemical analysis to identify the compounds present in the oil. Approximately 17 compounds were identified, of which 13 were identified by gas chromatography. The authors characterized monoterpene limonene as the major constituent of the essential oil, equivalent to approximately 70.75% of the total product. Moreover, the presence of β -pinene was also detected at a concentration of 13.19%. The antimicrobial profile of the two major isolated compounds resulted in MICs of 75 μg/mL and 500 μg/mL for limonene and β-pinene, respectively. Thus, the results were able to attribute limonene as the main compound responsible for the anti-*Helicobacter pylori* activity.

## Conclusion

This review demonstrates the grandiosity of the use of compounds derived from natural products against *Helicobacter pylori* and denotes the need for scientific and technological expansion regarding these agents. Future work focused on promoting a therapeutic arsenal endowed with significant pharmacological actions and low toxicity and cumulative effects is required.
